# Herbal compound triptolide synergistically enhanced antitumor activity of amino-terminal fragment of urokinase

**DOI:** 10.1186/1476-4598-12-54

**Published:** 2013-06-08

**Authors:** Yuli Lin, Nana Peng, Jianping Li, Hongqin Zhuang, Zi-Chun Hua

**Affiliations:** 1 The State Key Laboratory of Pharmaceutical Biotechnology, College of Life Science, Nanjing University, 22 Han Kou Road, Nanjing 210093, PR China; 2 Changzhou High-Tech Research Institute of Nanjing University, Changzhou 213164, Jiangsu, PR China; 3 Jiangsu TargetPharma Laboratories Inc., Changzhou 213164, Jiangsu, PR China

**Keywords:** Amino-terminal fragment of urokinase, Triptolide, Synergism, Apoptosis, Cell migration, Cell cycle, Xenograft

## Abstract

**Background:**

Urokinase (uPA) and its receptor (uPAR) play an important role in tumour growth and metastasis, and overexpression of these molecules is strongly correlated with poor prognosis in a variety of malignant tumours. Targeting the excessive activation of this system as well as the proliferation of the tumour vascular endothelial cell would be expected to prevent tumour neovasculature and halt tumour development. The amino terminal fragment (ATF) of urokinase has been confirmed effective to inhibit the proliferation, migration and invasiveness of cancer cells via interrupting the interaction of uPA and uPAR. Triptolide (TPL) is a purified diterpenoid isolated from the Chinese herb *Tripterygium wilfordii* Hook F that has shown antitumor activities in various cancer cell types. However, its therapeutic application is limited by its toxicity in normal tissues and complications caused in patients. In this study, we attempted to investigate the synergistic anticancer activity of TPL and ATF in various solid tumour cells.

**Methods:**

Using *in vitro* and *in vivo* experiments, we investigated the combined effect of TPL and ATF at a low dosage on cell proliferation, cell apoptosis, cell cycle distribution, cell migration, signalling pathways, xenograft tumour growth and angiogenesis.

**Results:**

Our data showed that the sensitivity of a combined therapy using TPL and ATF was higher than that of TPL or ATF alone. Suppression of NF-κB transcriptional activity, activation of caspase-9/caspase-3, cell cycle arrest, and inhibition of uPAR-mediated signalling pathway contributed to the synergistic effects of this combination therapy. Furthermore, using a mouse xenograft model, we demonstrated that the combined treatment completely suppressed tumour growth by inhibiting angiogenesis as compared with ATF or TPL treatment alone.

**Conclusions:**

Our study suggests that lower concentration of ATF and TPL used in combination may produce a synergistic anticancer efficacy that warrants further investigation for its potential clinical applications.

## Background

Various proteolytic enzymes play important roles in tumour invasion and metastasis process. These proteases involve cathepsins, collagenases, plasmin, or plasminogen activators [1]. Urokinase-type plasminogen activator (uPA) and its receptor, uPAR, are important components of cell surface proteolysis used by tumour cells and capillary endothelial cells, and therefore play crucial roles in the establishment, metastasis and angiogenesis of most solid tumours [2,3]. uPA and uPAR are over-expressed in diverse human malignant tumours in contrast to the corresponding normal tissue [4]. There are several potential mechanisms underlying the promotion of uPA and uPAR to tumour growth and invasion. Firstly, binding of uPA to uPAR leads to activation of plasminogen to plasmin, and the plasmin in turn activates latent matrix metalloproteases (MMPs) to dissolve the components of extracellular matrix (ECM) and the basement membrane [5,6]. Secondly, the binding of uPA and uPAR can also activate multiple cell signalling molecules via some growth factor receptors, such as integrins and EGFR, and then stimulate cell mobility and growth [7,8]. Finally, uPA-uPAR system is implicated in tumour-associated angiogenesis [9,10]. All these crucial roles of uPA-uPAR system in tumour growth and metastasis make it an ideal candidate for targeted cancer therapy. Therapeutic molecules aimed at interrupting the interaction of uPA and uPAR may inhibit both tumour cell invasiveness and tumour-associated angiogenesis, thereby might be effective in cancer therapy. For example, the monoclonal antibody against uPA or uPAR has been confirmed effective to inhibit the proliferation, migration and invasiveness of cancer cells *in vitro*[11,12]. Another known antagonist inhibitor of uPA-uPAR is ATF, the amino-terminal fragment of urokinase which harbours an epidermal growth factor (EGF)-like domain and a kringle domain. ATF could efficiently inhibit angiogenesis and tumour invasion *in vitro* and *in vivo* by competing with uPA for binding to both endothelial and tumour cell surfaces [13-15].

The Chinese herb *Tripterygium wilfordii* Hook F (TWHF) has been used for centuries in the treatment of rheumatoid arthritis and several other autoimmune and inflammatory diseases [16-18]. Triptolide (TPL; C_20_H_24_O_6_), a diterpenoid triepoxide, is purified from TWHF, which has been found to possess potent immunosuppressive and anti-inflammatory properties [19]. The antitumor activity of TPL was first reported 40 years ago, when it was observed to induce cell apoptosis in leukaemia. TPL has since attracted much research interest [20]. TPL has been observed to inhibit the proliferation of several types of cancer cells *in vitro* and to reduce the growth and metastasis of tumours *in vivo*[21]. Results from *in vivo* studies indicate that TPL inhibits tumour xenografts in nude mice from several human cancer cell lines, including melanoma, bladder cancer, breast cancer, and gastric and colorectal carcinoma [22,23]. Not only can TPL inhibit tumour growth directly *in vitro* and *in vivo* but it can also be efficacious as an adjunct agent for enhancing the antitumor effects of chemotherapeutic or other cytotoxic agents [24-26]. However, the therapeutic potential of TPL is still limited due to its strong toxicity [27,28].

The combined inhibitory effects of TPL and other anticancer drugs on tumour cell growth were reported to be superior to the effects of these agents used singly [24,29]. Considering the antitumor activity of both ATF and TPL, we therefore hypothesized that the combination of TPL and ATF would enhance apoptosis in human solid tumour cells. The results presented in this study demonstrate that TPL and ATF combined treatment synergistically induces apoptosis in several human solid tumour cell lines through caspase-dependent pathway. In addition, combination of TPL and ATF at a low dosage eliminates the cytotoxicity of normal cells induced by the individual drugs at their effective concentrations. The combined treatment of TPL and ATF also show robust *in vivo* efficacy, which strongly suggests that TPL has potential in modulating and enhancing the apoptosis and anti-angiogenesis induced by ATF on human solid tumour cells, especially colon cancer, and the synergistic effects of their combination point to a more promising modality for treating colon cancer.

## Results

### ATF expression and purification

**Figure 1:**
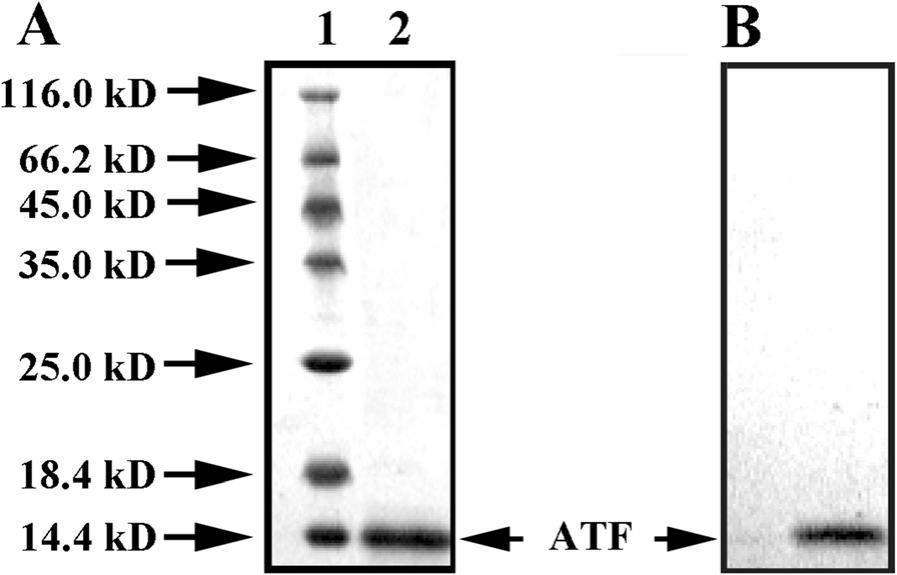
**Production and characterization of ATF.** (**A**) Purified ATF was analyzed by SDS-PAGE. Lane 1 protein Marker, ATF migrated around 15 kDa (Lane 2). (**B**) Identity of the protein was confirmed by Western blotting using poly-antibody against ATF.

The *Pichia* expression system was used to prepare ATF in soluble form. After ammonium sulphate precipitation, the target protein was concentrated in a small buffer volume and significant removal of some contaminants was achieved. In the ion exchange purification step, ATF was eluted as a single homogenous peak at 0.2 M NaCl. After the final step, the desired level of product purity (> 98%) was achieved. The final yield was about 18 mg/L culture. On SDS-PAGE, the mobility of the purified protein was found to correspond to a molecular weight of about 15 kDa (Figure 1A). The purified protein was further examined by Western blotting using anti-human ATF antibody. As shown in Figure 1B, the ATF migrated at 15 kDa as expected and no degradation was observed.

### Effect of single drug exposure on the growth of human HCT116 colon cancer cell line and A549 lung adenocarcinoma cell line

**Figure 2:**
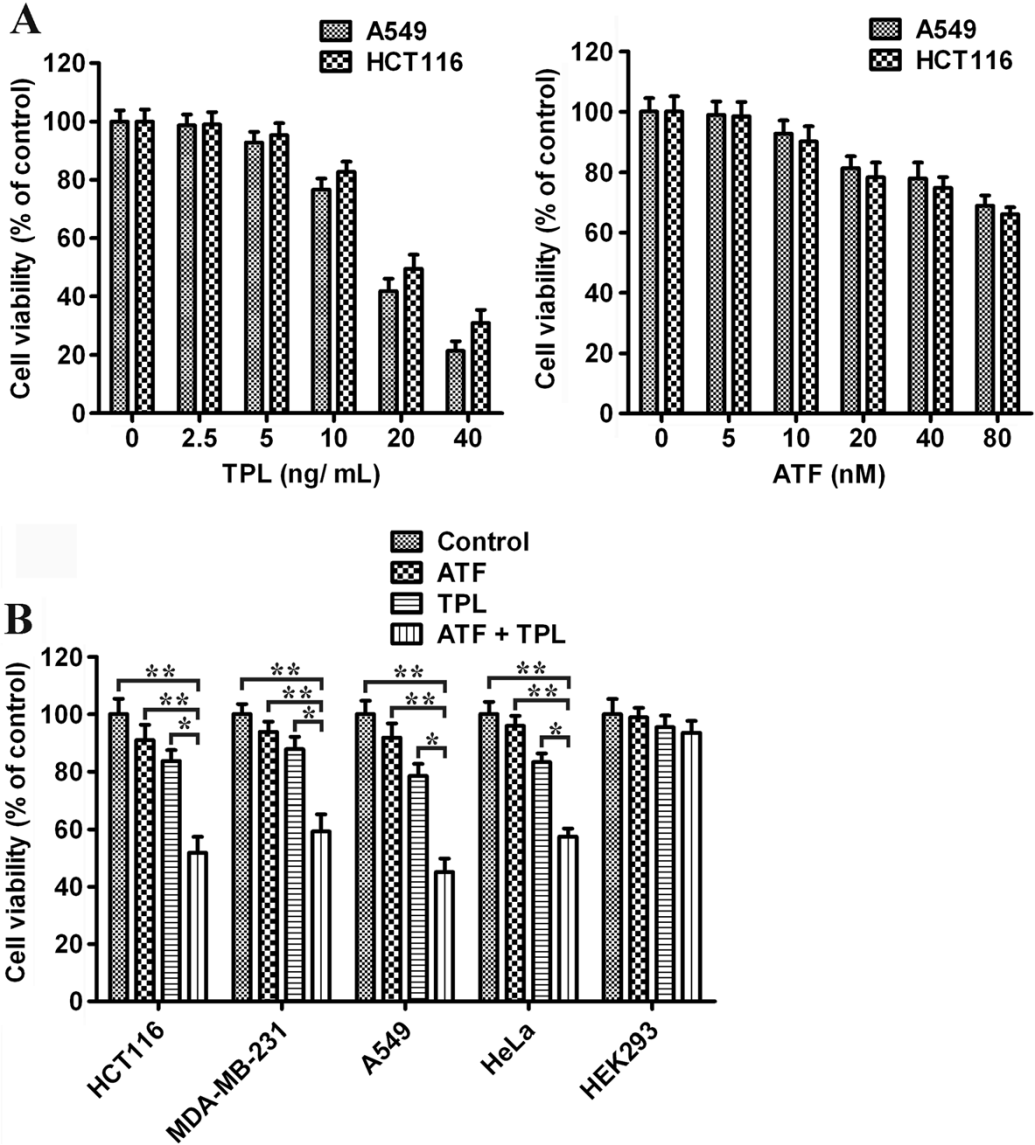
**Effect of TPL and ATF on the growth of tumour cells *in vitro*.** (**A**) Left, A549 and HCT116 Cells were treated with TPL at different concentrations (0, 2.5, 5, 10, 20, 40 ng/mL) for 24 h, and the cell viability was assessed by MTT assay. Right, A549 and HCT116 cells were treated with ATF at different concentrations (0, 5, 10, 20, 40, 80 nM) for 24 h, and the cell viability was assessed by MTT assay. (**B**) Combined effects of TPL and ATF on growth of HCT116, MDA-MB-231, A549, HeLa, and HEK293 cells. HCT116, MDA-MB-231, A549, HeLa, and HEK293 cells were exposed to TPL (10 ng/mL) and/or ATF (10 nM) for 24 h. Combined treatment resulted in significant growth inhibition of tumour cells, more than that by either drug alone; the degree of cytotoxicity in normal cells was not significant. *Each bar* shows the mean ± SD of three independent experiments, performed in triplicate. *p < 0.05, **p < 0.01.

The inhibition of proliferation by TPL and ATF of the human HCT116 colon cancer cell line and A549 lung adenocarcinoma cell line was assessed after 24 h of drug exposure, following 24 h culture in drug-free medium. As shown in Figure 2A, growth of the HCT116 and A549 cells was significantly inhibited in a dose-dependent manner *in vitro* (*p* < 0.05) by either drug treatment alone. For HCT116 cells, the inhibition ratio was 1.2 ± 0.24% at the concentration of 2.5 ng/mL of TPL, and 69.2 ± 1.65% at the concentration of 40 ng/mL. ATF at 5 nM had an inhibition ratio of 1.5 ± 0.42%, while the ratio was 34.2 ± 1.32% at 80 nM. In this study, we used the concentration at which ATF did not induce proliferation inhibition on its own. Therefore, in the subsequent combined treatment we choose ATF at the concentration of 10 nM and TPL at a low dosage of 10 ng/mL.

### Combined effect of TPL and ATF on growth of tumour cells

In order to assess the combined effect of TPL and ATF on tumour cell proliferation, MTT assay was performed. Four solid tumour cell lines and a normal cell line were treated with ATF (10 nM), TPL (10 ng/mL) or the combination for 24 hours. As shown in Figure 2B, ATF treatment alone did not cause obvious growth inhibition in all cell lines. TPL treatment alone induced 15-20% inhibition ratio, however, addition of ATF led to a significant increase in inhibition ratio (40-50%) as compared to TPL alone (*p* < 0.05) and to ATF alone (*p* < 0.01) in tumour cell lines. The combination index (CI) was 0.681 for HCT116 cells, 0.721 for MDA-MB-231 cells, 0.625 for A549 cells, and 0.721 for HeLa cells, indicating their synergistic effect on inhibiting the proliferation of tumour cells at lower concentrations. In contrast, no synergistic cytotoxicity was observed in HEK293 cells. These results showed that TPL at a subtoxic concentration had an enhanced effect on ATF-inhibited proliferation of tumour cells without increasing cytotoxicity to normal cells.

### Combined effect of TPL and ATF on tumour cell apoptosis

**Figure 3:**
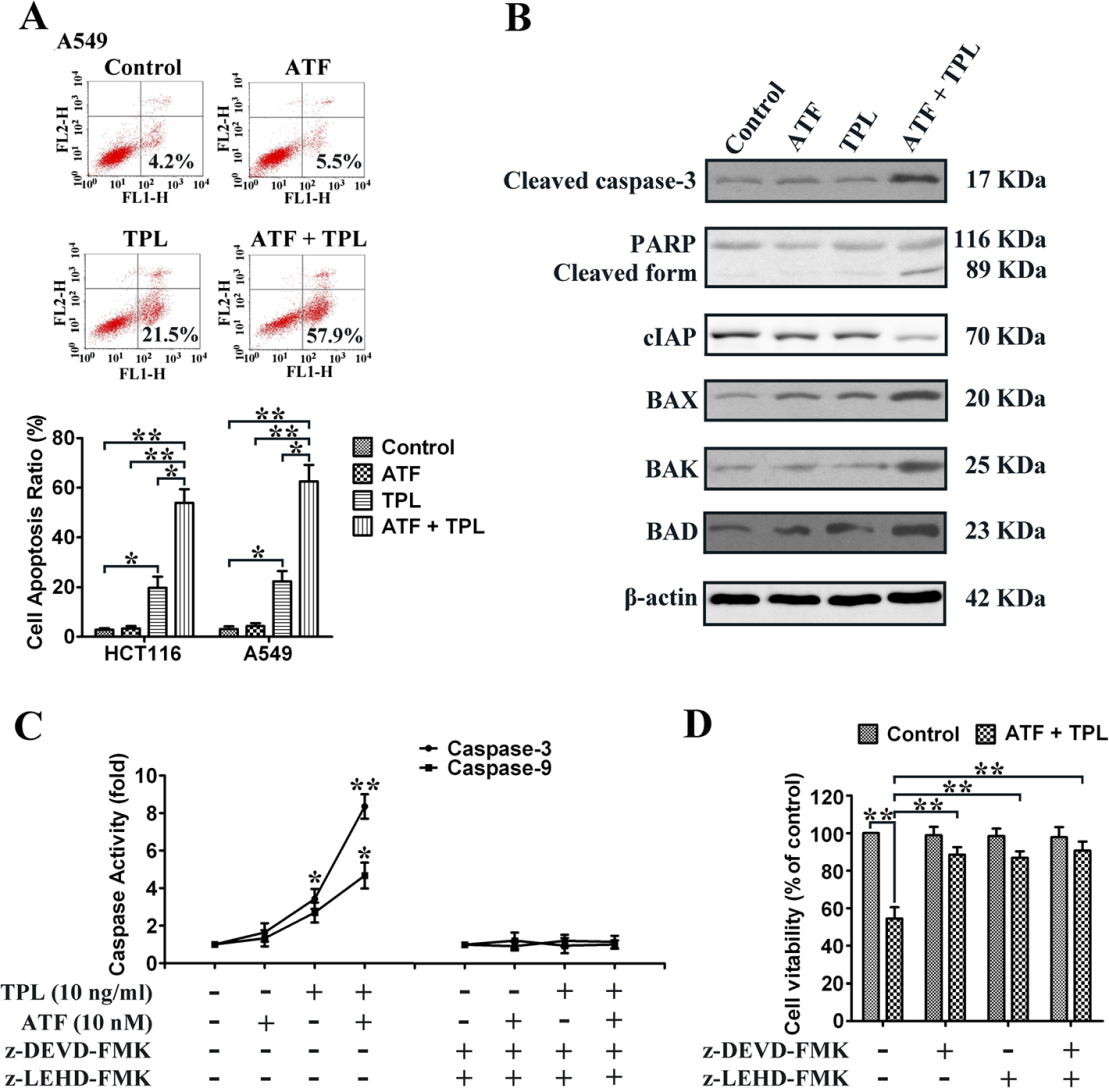
**Effects of TPL and ATF on tumour cell apoptosis and caspase activation.** (**A**) A549 and HCT116 cells were exposed to TPL (10 ng/mL) and/or ATF (10 nM). Twenty-four hours later, all cells were harvested for flow cytometry analysis. Annexin V/PI-stained cells were analyzed with the percentages of apoptosis cells. The experiments were carried out independently in triplicate; representative data are shown. *p < 0.05, **p < 0.01. Annexin V/PI double staining profile of A549 cells is also included. (**B**) Cleaved caspase-3, PARP, cIAP, BAX, BAD, and BAK expressions in HCT116 cells under different treatment conditions. (**C**) Activity of caspase-3 and caspase-9 in HCT116 cells treated with TPL and ATF alone or in combination for 24 h. Data are presented as fold increases as determined by quantitative analysis. (**D**) Viability of HCT116 cells after treatment with caspase inhibitors. Cells were treated with inhibitors for 2 h before the 24 h treatments, after which cell viability was determined by MTT assay. Data are representative of three independent experiments. *p < 0.05, **p < 0.01.

To determine whether tumour cellular viability decreased with TPL and ATF via apoptosis, we measured the externalization of phosphatidylserine on the cell membrane using Annexin V/PI staining. Two different solid tumour cell lines (HCT116 and A549) were exposed to ATF (10 nM), TPL (10 ng/mL) or a combination of both (ATF 10 nM + TPL 10 ng/mL). As shown in Figure 3A, after 24 h of treatment, ATF alone had no obvious effect on tumour cell apoptosis, while single therapy with TPL induced 15-25% apoptosis ratio. However, when HCT116 and A549 cells were exposed to combined therapy with TPL and ATF, the number of cells undergoing apoptosis significantly increased (55-65%). This effect was statistically significant as compared to single therapy with either drug alone.

### Regulatory mechanisms of TPL and ATF-induced apoptosis in HCT116 cells

To explore the mechanisms of TPL and ATF-induced apoptosis in HCT116 cells, activation of caspases and expression of pro-apoptotic proteins were analyzed by Western blotting assay. Caspases are the main enzymes that mediate apoptosis. Any stimuli that triggers apoptosis eventually leads to the activation of the effector caspases, including caspase-3, caspase-6, and caspase-7. In cells treated for 24 h, only the combined treatment with TPL and ATF significantly enhanced the cleavage of procaspase-3 and the downstream PARP, while treatment with TPL or ATF alone caused minimal proteolytic processing of procaspase-3 and cleavage of PARP. In addition, combined treatment of HCT116 cells with TPL and ATF noticeably increased the levels of BAX, BAK and BAD with a prominent reduction of cIAP level (Figure 3B). Caspase activity, shown in Figure 3C, indicated that caspase-3 and caspase-9 activities were elevated to 1.6- and 1.3-fold over controls in cells treated with ATF and 8.5- and 4.7-fold over that in combined treatment, respectively. Co-treatment with the caspase inhibitors z-DEVD-FMK and z-LEHD-FMK abolished caspase activation induced by TPL and ATF and rescued HCT116 cells from treatment-induced cell death (Figure 3C and 3D). Cell viability was also increased by caspase inhibitors after combined treatment. These findings indicate that activation of a caspase-involved apoptotic pathway is one of the major mechanisms via which TPL exerts its synergistic effect on ATF-treated HCT116 cells.

**Figure 4:**
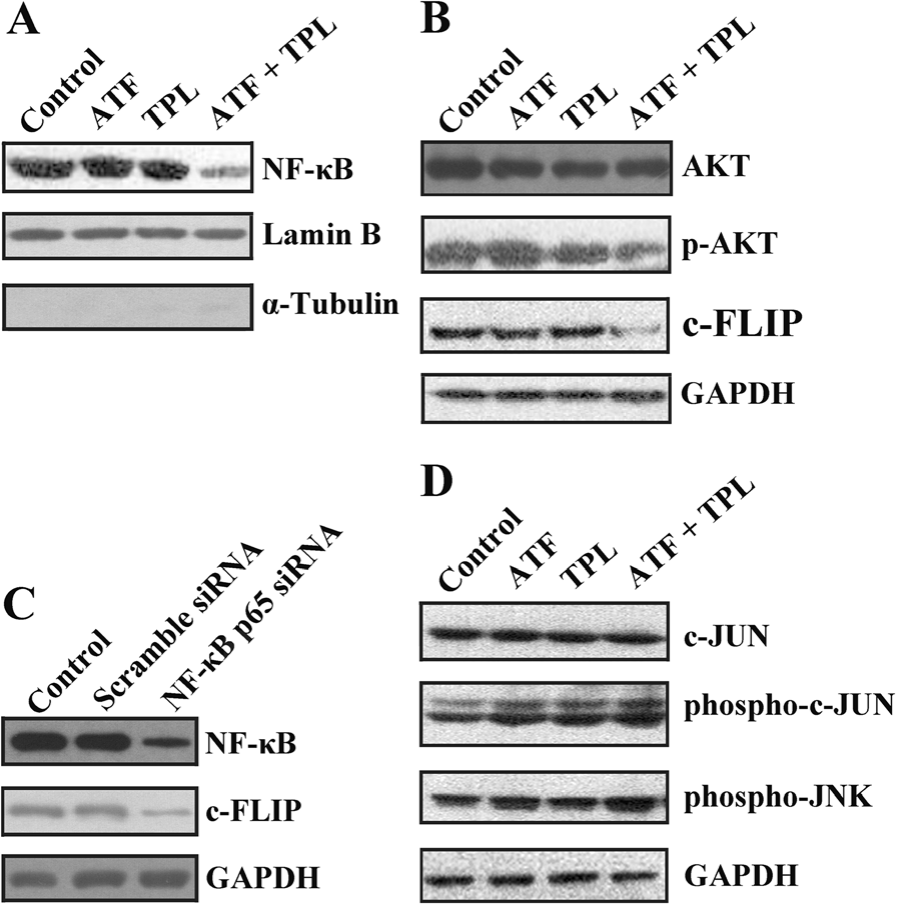
**Effects of TPL and ATF on NF-κB and JNK signalling pathway.** (**A**) HCT116 cells were treated with TPL and ATF alone or in combination for 24 h. Nuclear proteins were extracted and subjected to Western blotting for p65 detection. Lamin B was used as loading control and α-tubulin was used as a cytoplasmic protein marker to rule out cytoplasmic contamination in the nuclear fraction. (**B**) HCT116 cells were treated with TPL and ATF alone or in combination for 24 h. AKT, p-AKT, and c-FLIP proteins in whole cell lysates were determined with specific antibodies. GAPDH was used as loading control. (**C**) HCT116 cells were transfected with scramble siRNA or NF-κB p65 siRNA. 48 h later, cell extracts were applied for Western blotting analysis using NF-κB p65 and c-FLIP antibodies. (**D**) HCT116 cells were treated with TPL and ATF alone or in combination for 24 h. c-JUN, p-c-JUN and p-JNK protein in whole cell lysates were determined with specific antibodies.

The cooperation of TPL with chemotherapy and cytokines to induce apoptosis in cell lines has been attributed to inhibition of the NF-κB pathway [30]. Thus, we investigated whether TPL at the dosage of 10 ng/mL was able to modify the rate of NF-κB inhibition. Low dosage of ATF or TPL alone had no obvious effect on the expression of NF-κB/p65. However combined treatment decreased the level of NF-κB/p65 in the nucleus of HCT116 cells co-treated for 24 h (Figure 4A). c-FLIP, one of the targeted genes of NF-κB, is known to interfere with caspase activation downstream of death receptors. To evaluate the combined effect of ATF and TPL on c-FLIP expression, we treated HCT116 cells with ATF in the absence or the presence of TPL. Our Western blotting assay showed that combined treatment decreased c-FLIP expression, while ATF or TPL alone had no effect on c-FLIP expression (Figure 4B). To further determine whether NF-κB inhibition resulted in reduction of c-FLIP, HCT116 cells were transfected with NF-κB p65 siRNA. Western blotting analysis revealed that siRNA against NF-κB p65 effectively reduced NF-κB/p65 and c-FLIP-L levels in the transfected cells (Figure 4C). AKT was reported to suppress apoptosis by stimulating the transactivation potential of the RelA/p65 subunit of NF-κB [31,32]. Therefore, the detection of Ser473 p-AKT and total AKT in HCT116 cells was performed following exposure to TPL and ATF for 24 h. Figure 4B revealed that the phosphorylation level of AKT was markedly decreased after co-treatment with TPL and ATF, but not either drug alone. JNK is known to promote apoptosis by many cellular stresses, including oxidative stresses, and DNA-damaging agents [33] and plays important roles in cell proliferation and apoptosis [34]. We hypothesized that JNK might be activated by cellular stress induced by TPL and ATF combined treatment. As expected, the level of phospho-JNK increased in cells co-treated with TPL and ATF. Furthermore, the combination of TPL and ATF induced a slight increase in the level of phospho-c-JUN in HCT116 cells (Figure 4D). In contrast, low dosage of ATF or TPL alone failed to activate the JNK-c-JUN pathway. Taken together, these findings suggest that TPL and ATF cooperatively induce apoptosis through the suppression of NF-κB transcriptional activity, subsequently reduction of c-FLIP expression, and activation of caspases-9/caspase-3 and the JNK-c-JUN pathway.

### TPL and ATF combined therapy initiated cell cycle arrest at S phase in HCT116 cells

**Figure 5:**
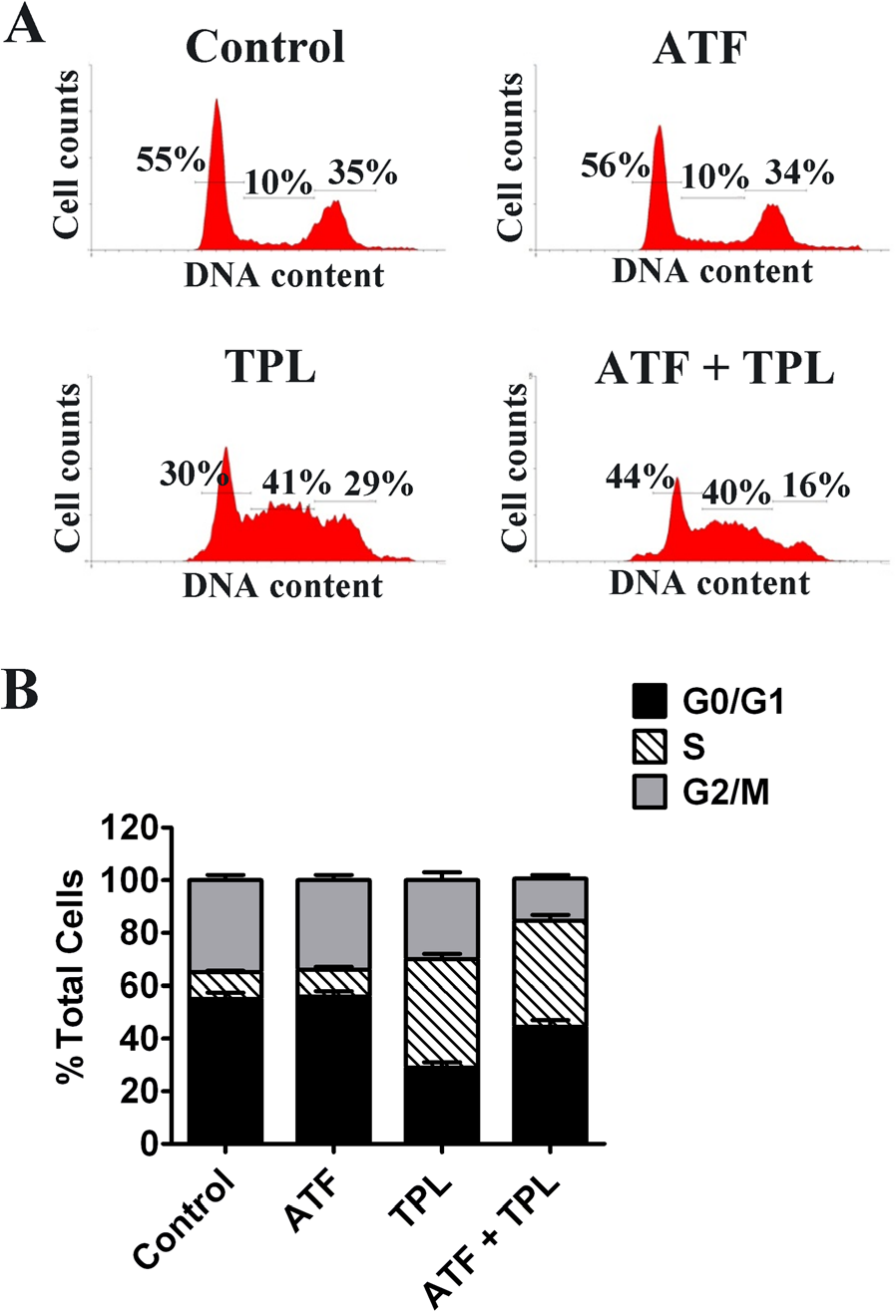
**Effect of TPL and ATF on cell cycle distribution.** (**A**) HCT116 cells were treated with TPL (10 ng/mL) and/or ATF (10 nM). Twenty-four hours later, cells were fixed and stained with PI for flow cytometry as described in Materials and Methods. DNA histograms were modeled with CellQuest analysis software. The experiments were carried out in triplicate and representative data are shown. (**B**) Phase percentages for G0/G1, S, and G2/M are depicted by bar graph. Data represent mean values of triplicate samples.

TPL has been reported to have the ability of inhibiting cell proliferation to execute its antitumor effect. Thus, we detected the effect of ATF, TPL or the combination on cell cycle distribution. As shown in Figure 5, ATF treatment alone had no effect on cell cycle distribution. However, when cells were incubated with TPL, the cell population of G0/G1 phase decreased from 55.3% to 29.8% and S phase increased from 10.3% to 41.2%. When combined with ATF, the cell population of S phase was similar to TPL treatment alone with the ratio of 40.5%, and the cell population of G2/M phase, an indicator of cellular mitosis or cell division, dropped from 30.4% to 16.2% as compared to TPL single treatment. The decrease in G2/M phase during the combination therapy was due to the increased cell cycle arrest in G0/G1 phase (from 29.8% for TPL single treatment to 43.6% for TPL and ATF combined treatment). These results indicate that the major effect of TPL on cell cycle is S phase arrest, and ATF can reinforce the cell proliferation inhibition effect of TPL by endowing with additional ability of G0/G1 cell cycle arrest.

### Combined effect of TPL and ATF on HUVEC and HCT116 cell migration

**Figure 6:**
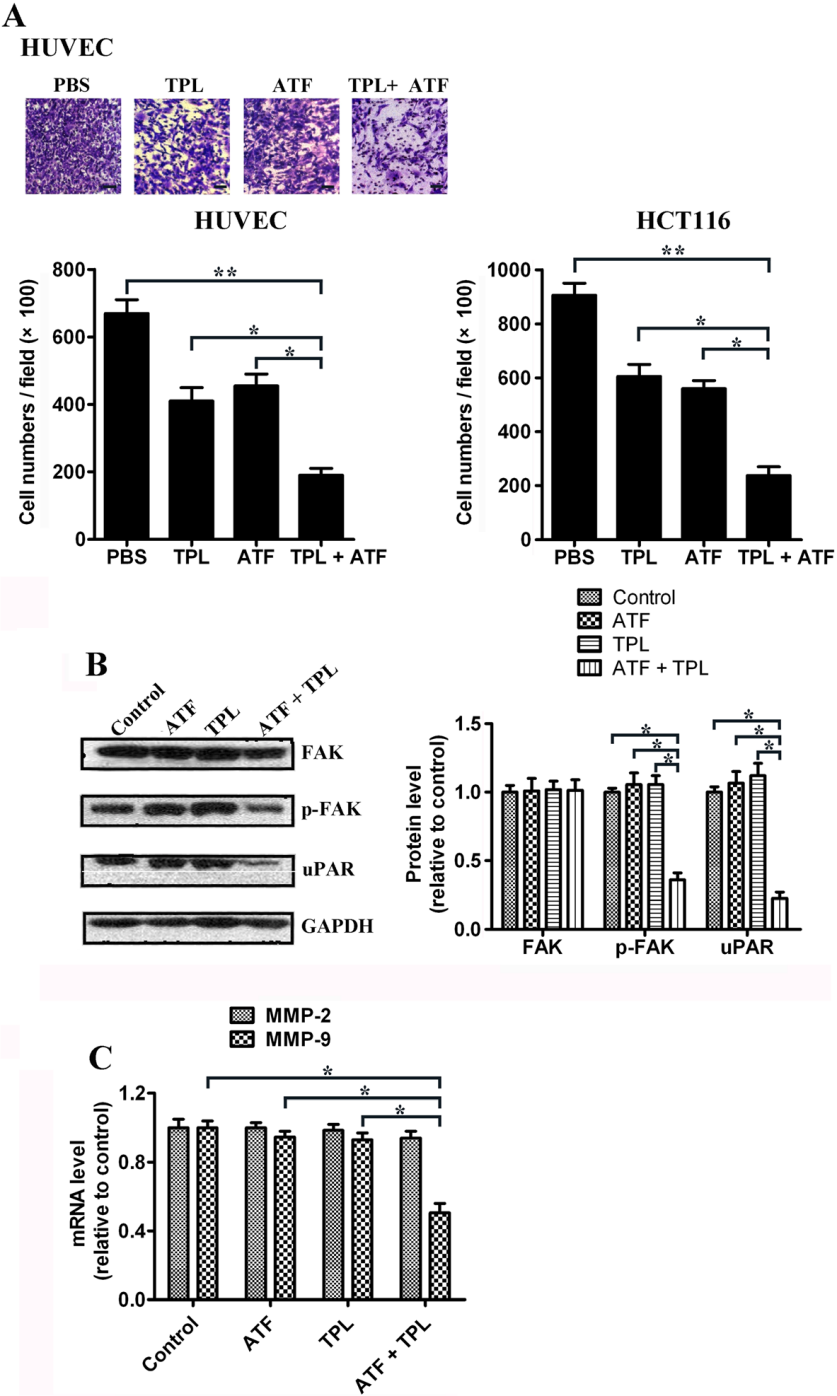
**Effect of TPL and ATF on cell migration.** (**A**) Transwell assay. HUVECs and HCT116 cells were treated with TPL (10 ng/mL) and/or ATF (10 nM). After 16 h pretreatment and 9 h incubation in the upper chamber, the cells migrating to the lower membrane were stained and counted in five fields with a magnification of × 100. N = 3, bar = 50 μm. The experiments were carried out in triplicate and representative data are shown. (**B**) Effects of TPL and ATF on uPAR, FAK and p-FAK protein expression. HCT116 cells were treated with TPL (10 ng/mL) and/or ATF (10 nM). Twenty-four hours later, cells were harvested for Western blotting analysis using indicated antibodies. The level of GAPDH served as the loading control. Band intensities were calculated using software Image J. Relative intensities are also shown. Data represent mean values of triplicate samples. (**C**) HCT116 cells were treated with TPL (10 ng/mL) and/or ATF (10 nM). Twenty-four hours later, cells were harvested for RNA extraction and quantitative real time PCR using primers specific for human MMP-2, MMP-9, and GAPDH (internal control). Data represent mean values of triplicate samples. *p < 0.05, *p < 0.01.

In order to precisely characterize the effect of TPL and ATF on endothelial cell and tumour cell migration, serum stimulated haptotaxis motility, measured by the transwell motility chamber assay, was used to examine the effect of TPL and ATF on HUVEC and HCT116 cell migration. As shown in Figure 6A, the cells migrating to the lower membrane were stained and quantified. We found that, at a low dosage, ATF or TPL alone showed slight inhibition of cell migration. However, combined treatment with TPL and ATF showed more significant inhibition of cell migration than single therapy alone, which reduced the migration of HUVECs by 71.6% or 58.2% compared with control PBS group or ATF group, respectively. Similar results were also obtained in HCT116 cells. As we know, uPAR-dependent cell signalling events impact cell migration and survival. To explore the mechanisms underlying TPL and ATF combined effect on cell migration, Western blotting analysis was further accessed to determine the protein expression level of FAK and uPAR, which have been demonstrated to play important roles in cell migration. The results indicated that combined treatment with TPL and ATF significantly decreased phosphorylation level of FAK, while total FAK protein remained unchanged. In contrast, TPL or ATF alone had no effect on the phosphorylation of FAK. Similar results were observed in uPAR protein expression. Decreased expression level of uPAR was found in co-treated cells, compared with ATF or TPL treatment alone (Figure 6B).

uPA/uPAR system was reported to induce MMPs activity in cancer cells and then promote cancer cell migration and metastatic potential [35]. Previous reports suggested that down-regulation of uPAR decreased the expression of MMP-2 and MMP-9 [36]. Consistently, our qPCR results showed that combined treatment with TPL and ATF decreased the mRNA level of MMP-9 in HCT116 cells. However, no obvious inhibitive effect on mRNA expression of MMP-2 was found in cells co-treated with TPL and ATF (Figure 6C).

### Combination of TPL and ATF retarded the development of colon cancer xenografts in nude mice

**Figure 7:**
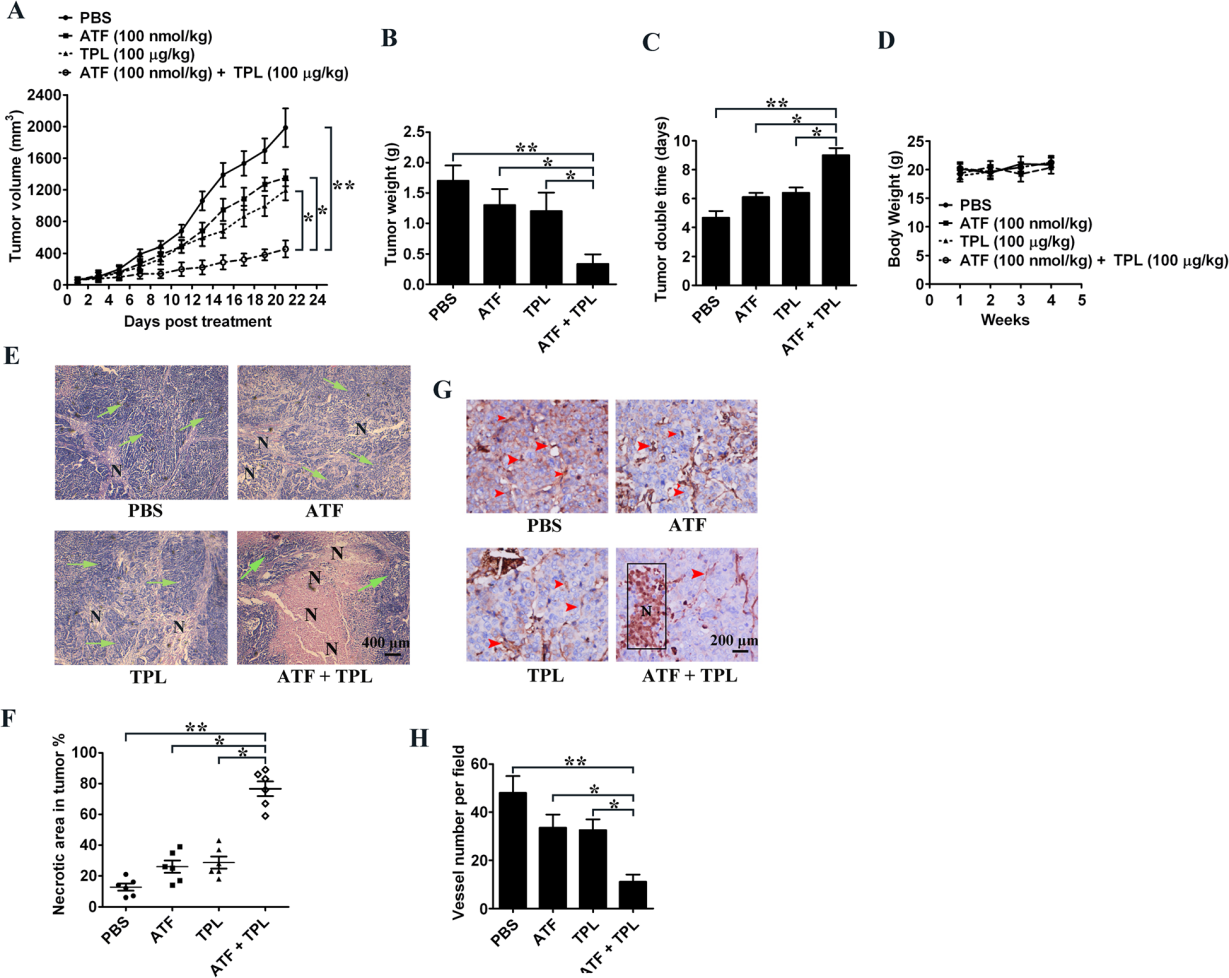
**The antitumor effect of TPL and ATF on the xenograft of colon cancer.** HCT116 cells were injected subcutaneously into the dorsal flanks of athymic nude mice. When tumors reached a size of approximately 50 mm^3^, mice were i.p. with ATF and TPL or the combination every two day for a total of 21 day. (**A**) The tumor growth inhibitory effects of different treatments were compared. (**B**) At the end of the study, the excised tumors from each group were weighed. (**C**) Tumour double time of each group. (**D**) The weight of nude mice from each group did not change significantly during the experiment. (**E**) Determination of tumor necrosis after combined treatment with TPL and ATF. Tumour necrosis areas are shown by H&E staining and observed under light microscope (× 100). The viable tumor cells are indicated by a green arrow. (**F**) Quota of tumour necrosis. Tumour necrosis was determined by software Image J (NIH, USA). Two sections/mouse and three mice were prepared. (**G**, **H**) Blood vessel density within tumour was characterized by anti-CD31 immunostaining using anti-mouse CD31 monoclonal antibody (**G**) and determined by the average number of vessels in 3 regions of highest density at × 200 magnifications in each section (assay was repeated in 4 sections per mouse and 3 mice were tested) (**H**). All data are shown as mean ± SD. *p < 0.05, **p < 0.01.

The antitumor effect of TPL in combination with ATF was analyzed in a xenograft tumour model by transplanting HCT116 cancer cells into athymic nude mice. On the 7th day post-implantation, mice were randomly divided into 4 groups before the tumour was palpated, with at least 8 tumour-bearing mice in each group. Tumour volume was significantly reduced after intraperitoneally injection of TPL and ATF for 21 days as compared to TPL or ATF Monotherapy (Figure 7A). Both TPL and ATF monotherapy also inhibited the growth of xenograft tumours to some extent, but the effects were not as significant as those seen in the combined treatment group. At the end of the study, we removed the tumours and measured their weight for each group. Combined treatment with TPL and ATF clearly reduced tumour weight compared with the control group, ATF or TPL single treatment (Figure 7B). Tumour doubling time was prolonged from 4.67 days in mice receiving PBS, 6.12 days in mice receiving ATF, 6.43 days in mice receiving TPL to 9.05 days in mice receiving TPL + ATF (*p* < 0.05; CI = 1.36; Figure 7C), indicating a supra-additive or synergistic effect of TPL and ATF. In addition, no significant change in body weight was observed in mice treated with TPL alone, ATF alone, or TPL and ATF combined treatment (Figure 7D), indicating that there is no obvious toxicity for all of the treatment regimens. Furthermore, light microscopy revealed that tumour tissues in mice receiving TPL and ATF displayed more severe necrosis than control or TPL or ATF single therapy (Figure 7E). The percentage of necrotic area in tumours increased from 12.7% in mice receiving PBS, 26.2% in mice receiving ATF, 28.7% in mice receiving TPL to 76.4% in mice receiving TPL + ATF (Figure 7F). TPL and ATF single therapy or untreated control displayed tissue necrosis interspersed with viable tumour cells, whereas TPL and ATF combined treatment induced large areas of continuous necrosis within tumours (Figure 7E). Immunohistochemical studies further revealed that TPL and ATF combined treatment markedly reduced the expression of CD31, a marker of neoangiogenesis (Figure 7G and 7H), suggesting that combination of TPL and ATF could inhibit tumour progression primarily through suppressing tumour-related angiogenesis.

## Discussion

Colon cancer remains a major public health threat and accounts for approximately 13% of all cancers [37]. More effective treatments and earlier detection have led to improved survival over recent decades. However, around 50% of newly diagnosed colon cancer patients will eventually progress due to micro metastases, and die of their disease, in spite of the advances in surgical techniques and radiotherapy. Therefore chemotherapy becomes one of the most important means of extending the survival of colon cancer patients. The development of cancer involves a complex interplay among cellular processes, and treatment with a single agent is rarely effective. Combination therapy is now considered to be a standard approach to chemotherapy [38]. There are many advantages to combination therapy, including the targeting of multiple critical molecular processes, delivery of lower dose agents with lower toxicity, and increased patient tolerance. The effectiveness of combination chemotherapy has stimulated an interest in exploring drugs with different modes of activity at lower dosages [39].

The coordinated interaction of different proteolytic systems is important for tumour cell invasion and metastasis [40]. The invasive capacity of tumour cells can be suppressed by synthetic inhibitors against various proteases or by plasminogen activator system antagonists. The uPA system has pivotal roles in tumour growth, angiogenesis, and metastasis [41]. The binding of uPA to uPAR has been shown to mediate various other signalling cascades [42], although the role of these cascades in tumour progression is poorly understood. Since the uPA-uPAR system contributes to the invasion and motility of several cell types associated with tumour progression, the inhibition of the uPA-uPAR interaction may have significant antitumor effects. ATF, the amino terminal fragment of urokinase, has been demonstrated to act as an angiostatic molecule that targets the uPA-uPAR system and inhibits cell invasion and migration. By blocking the attachment of uPA to uPAR, ATF could effectively shut down the plasmin activation on the surface of both tumour and activated endothelial cells, which is essential for angiogenesis-related ECM degradation, new blood vessel formation, and accordingly the invasive phenotype of primary tumours [14,15]. In a recent study, the invasiveness of a highly metastatic human lung giant-cell carcinoma cell line transfected with ATF cDNA was significantly inhibited *in vitro*, as was the lung metastasis of implanted cells in a spontaneous metastasis model [43]. Li *et al*. also showed that adenovirus-mediated delivery of ATF suppressed growth of xenografted MDA-MB-231 human breast cancer cells grown in athymic mice [44]. These data suggests that ATF is a good candidate for cancer therapy. Nevertheless, the clinical experience and treatment of other solid tumours tell us that only a few solid tumours respond to single agent-based therapy. Chronic exposure to chemotherapeutic agents can induce the selection of clones that are resistant to that particular agent; therefore, overtime tumour resistance can occur. Additionally, solid organ tumours sometimes have intrinsic resistance to the drug before any treatment has started. It cannot be overemphasized that the probability that drug resistance develops over the course of the disease decreases if different agents are combined. Combined therapy also allows decreasing drug doses, decreasing the likelihood of toxicity.

TPL, a natural, active compound isolated from *Tripterygium wilfordii* Hook F, is known to induce apoptosis in several cancer cell types by activating both the extrinsic and intrinsic pathways of apoptosis in tumours [21]. As a promising immune-suppressor, TPL has been widely used in Chinese medicine. TPL has multiple pharmacological activities, including anti-inflammatory, immunosuppressive, male anti-fertility and anticancer effects [45,46]. Research into its mechanisms of action has revealed that it potently inhibits monocyte activation, activates caspases and other pro-apoptotic signalling cascades, inhibits angiogenesis and reverses drug resistance [47,48]. Recent studies demonstrate that TPL also possesses anti-cancer activity and inhibits cancer cell proliferation *in vitro* and *in vivo*[21]. Although TPL alone was very effective to kill tumour cell lines, it is not curative and the safe dose range for *in vivo* application is relatively narrow. A major concern about using TPL for clinical antitumor applications is its toxicity. Shamon *et al*. [49] reported that TPL exerted a modest antitumor activity when administered at a dose of 25 μg/mouse 3 times per week intravenously to nude mice carrying human breast tumors, but higher doses (50 μg/mouse) were lethal, suggesting a narrow therapeutic window of TPL treatment. Severe side effects happened in a recent phase I clinical study using F60008 [50], which is a semi-synthetic derivate of TPL, in patients with solid tumours. In preliminary study, we found that i.p. administration of 100 μg/kg doses of TPL exerted slight antitumor effects, and that the mice treated with 100 μg/kg TPL did not show any obvious side effects. However, weight loss, skin inflammation and vessel inflammation were observed in the mice treated with 400 μg/kg TPL, and higher doses of TPL displayed stronger effects but the side effects were more severe (data not shown). Therefore it would be much more beneficial if it can be used at a relatively lower dose to sensitize the cytotoxicity of other anti-cancer drugs. TPL has been shown great value when used in combination with other antitumor treatments, inducing higher levels of cell death by increasing tumour cell sensitivity to chemotherapy or radiation. Previous studies indicate that TPL can efficiently enhance the cytotoxicity of some cytokines and anti-cancer drugs [24,51,52].

**Figure 8:**
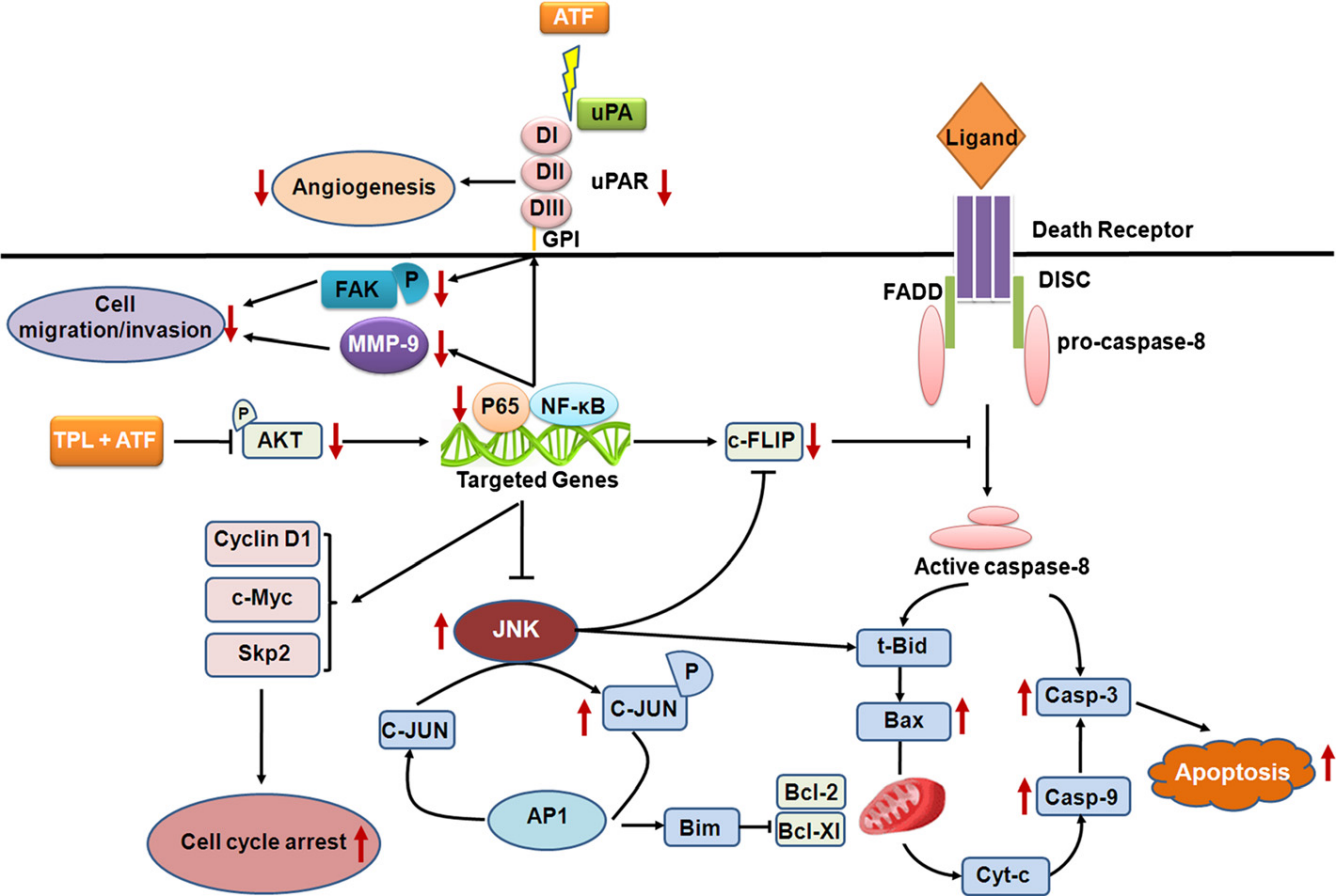
**A working model for the synergistic effect of TPL and ATF on tumour cells.** TPL and ATF cooperatively induce apoptosis through caspase-dependent pathway and cell cycle arrest. In HCT116 cells, combined treatment with TPL and ATF at a low dosage inhibits AKT phosphorylation and subsequently inactivates NF-κB. The inhibition of NF-κB leads to down-regulation of c-FLIP, activation of caspases-9/caspase-3 and JNK-c-JUN pathway, and cell cycle arrest, which finally promotes cell apoptosis. In addition, the suppression of NF-κB leads to decreased expression of uPAR, MMP-9 and phospho-FAK, thereby inhibiting tumor cell migration and invasion. Furthermore, ATF can segregate uPA from its receptor uPAR, thus blocking ECM remodeling and anti-angiogenesis processes.

Since both ATF and TPL exhibit antitumor activity, we formulated the hypothesis that combined therapy with these two drugs increases the effectiveness as compared with single therapy. In this study, we tested the *in vitro* and *in vivo* enhancing effect of TPL on the cytotoxicity of ATF in a panel of solid tumour cell lines. Using MTT assay we found that TPL inhibited the growth and proliferation of ATF-treated tumour cells synergistically. Compared to TPL or ATF alone, low dosage of these two drugs in combination induced substantial apoptosis of tumour cells. Cell apoptosis is known to be programmed and finally executed by caspase-3, through several signalling pathways involved in apoptosis regulation. To further exploit the antitumor mechanism of TPL and ATF, we detected the activation of caspase-9, caspase-3 and NF-κB/p65. Our results indicated that induced apoptosis of HCT116 cells by the combination of TPL and ATF was mediated through caspase-9/caspase-3 activation and NF-κB/p65 inhibition. In turn, caspases activation led to PARP cleavage, DNA damage and fragmentation, nuclear condensation, and eventually, the induction of apoptosis. NF-κB/p65 that comprises a heterotrimer of p50 and p65 binds to its inhibitory protein IκBα, thereby leading to the release of the p50-p65 heterodimer, which then translocates to the nucleus and associates with the promoter regions of multiple target genes. In this study, we found that TPL and ATF combined treatment can down-regulate NF-κB/p65 protein expression and this finding is consistent with that of other reports [53,54]. NF-κB is generally considered to be a survival factor that activates expression of various anti-apoptotic genes, e.g. Bcl-2, Bcl-xL, Mcl-1 and c-FLIP that block apoptosis [55]. Inhibition of NF-κB will lead to down-regulation of the NF-κB-regulated anti-apoptotic proteins, thereby promoting apoptotic cell death. Indeed, combined treatment with TPL and ATF significantly decreased the expression of c-FLIP, a well-known anti-apoptotic protein, and finally led to cell apoptosis. It has been reported that the ser/thr kinase AKT can promote NF-κB activity [31,32]. In the current study, we found that TPL and ATF combined treatment did not affect the total expression of AKT, but significantly decreased the phosphorylation level of AKT (Figure 4B). The inactivation of AKT may lead to transcriptional inhibition of NF-κB, and the previously well-characterized down-regulation of c-FLIP expression by inactivated NF-κB. In addition, co-treatment with TPL and ATF also led to JNK activation. The activation of JNK promotes apoptosis in a manner that is dependent on the cell type and the context of the stimulus. In the past, the contributions of the NF-κB and JNK pathways to cell death have been discussed independently. However, recent studies have indicated that one of the anti-apoptotic functions of NF-κB is to down-regulate JNK activation [56]. Therefore, we speculated that TPL and ATF in combination could activate JNK in tumor cells through inactivating NF-κB, thus contributing to apoptosis. In addition, the activation of JNK is also involved in the down-regulation of c-FLIP-L [57]. Thus, the inhibition of NF-κB, up-regulation of JNK activity and subsequently reduction of c-FLIP expression might contribute to the increased sensitivity to TPL and ATF-mediated apoptosis (Figure 8).

Cell cycle regulation is closely linked to cell proliferation, and one of the notable features of a tumour is abnormal cell cycle management. TPL has previously been shown to induce cell accumulation in the S phase [52,58]. Cell apoptosis seems like to be closely associated with the cell cycle arrest in S phase via accelerating cells into S phase and hampering cells out from S phase. In human colon cancer cells, we confirmed that TPL accumulated cells in S phase and thus caused cell apoptosis. When in combination with ATF, cells were maintained the S phase arrest and the population of cells in G2/M phase was decreased as compared to TPL single therapy. Nevertheless, some researches demonstrated that TPL treatment triggered a G0/G1 cell cycle arrest and apoptosis in gastric cancer and multiple myeloma cells [19,59]. In our study, treatment with TPL alone caused S phase arrest, not G0/G1 arrest in HCT116 cells. However, ATF could help TPL to restore its ability of G0/G1 arrest, meanwhile retain its S phase arrest. Taken together, these results indicate that the cell cycle phase arrest TPL will induce is cancer cell type-specific and ATF can enhance the cell cycle arrest ability of TPL, thus eventually increasing cell apoptosis. In addition, NF-κB also plays a crucial role on cell cycle by regulating key proto-oncogenes, including Cyclin D1, c-Myc and Skp2 [60]. Thus, the inhibition of NF-κB may partially contribute to cell cycle arrest by combined treatment with TPL and ATF (Figure 8).

Cell motility is one of the prerequisites for the invasion and metastasis of malignant tumours. Most cancer patients do not die from local complications of their primary tumour growth, but rather from the development and spread of the tumour. Therefore, metastasis is one of hallmarks of malignant tumour and a major cause of death among cancer patients. Several reports have indicated that TPL can reduce the growth and metastasis of tumours *in vivo* and *in vitro*, via inhibition of heat shock protein 70 (HSP70), CXC chemokine receptor 4 (CXCR4), or uPAR [61-63]. In this study, we found that, in the presence of ATF at a low concentration, the motility of tumour cells was decreased, which clearly demonstrated that ATF alone could partially inhibit this step. When combined with TPL, the inhibition of tumour cells migration was significantly enhanced. Mohanam *et al*. reported that a glioma cell line over-expressing ATF exhibited impaired adhesion, motility and colonization, the mechanism underlying those phenotypes was the rearrangement of cytoskeleton [15]. Cell motility is made up with successive attachment and detachment. Upon the binding of uPA, uPAR is subjected to directly interacting with vitronectin, and thereby improved the cell adhesion and attachment [64,65]. In the presence of PAI-1, the complex containing uPA-PAI-1-uPAR will be engulfed by cell, accompanied with the degradation of uPA-PAI within lysosome and the recycling of intact uPAR to cell surface. This process may induce the occurrence of cell detachment [66]. Presumably, ATF slows the movement by impairing the recycling of uPAR on the cell surface. Unlike uPA, ATF is incapable of binding PAI-1 [66,67], which blocks the uPAR recycling and attenuates the attachment-detachment cycle. Therefore, cells overexpressing uPAR may adapt to be quiescent upon the ATF binding. To further clarify the mechanisms underlying combined effect of TPL and ATF on cell migration, we examined the uPAR-dependent signalling pathway. We found that, combined treatment with TPL and ATF led to inhibition of uPAR and FAK phosphorylation significantly. Specifically, treatment of HCT116 cells with ATF or TPL alone did not affect the expression level of uPAR protein and downstream FAK phosphorylation, thus indicating that the inhibition of cell migration was not an additive but indeed a cooperative effect of TPL and ATF. It is reported that TPL inhibits uPAR expression via blocking NF-κB signalling [63]. Thus, we speculated that low dosage of TPL and ATF in combination led to inhibition of NF-κB, which finally down-regulated uPAR expression. Furthermore, inhibition of NF-κB pathway may also down-regulate uPA [68]. In mammary tumour cells, uPA binding to uPAR activates FAK through a still unknown partner molecule [69]. Thus, the down-regulation of uPA and uPAR may lead to subsequent decreased phosphorylation level of FAK. Taken together, these results provide evidence that TPL and ATF combination-triggered inhibition of cell migration is probably mediated through NF-κB-uPA/uPAR-FAK-dependent cell signalling pathways (Figure 8). Additionally, we found that combined treatment of ATF and TPL decreased the mRNA level of MMP9 but not MMP2 in HCT116 cells, which are mainly involved in the metastasis process. On the contrast, ATF or TPL single therapy had no obvious effect on MMP-9 expression, indicating that the expression of MMP-9 is synergistically regulated by TPL and ATF. Although MMP-9 shares fairly broad substrate specificity and structure features with MMP-2, both enzymes differ considerably in terms of transcriptional regulation. The 5′ flanking sequence of MMP-9 gene harbors NF-κB binding sites, while the expression of MMP-2 is mainly regulated by SP-1 [70]. The combined effect of TPL and ATF on MMP-9 expression is probably through NF-κB inhibition. These could be presumed to be one of the reasons for different effect of TPL and ATF on the gene expression of MMP-9, compared with MMP-2.

uPA-uPAR system plays a crucial role in the ECM degradation and remodelling in the process of angiogenesis, thereby may affect the formation of neovessel structure and the tumour development [3]. In the *in vivo* tumour model experiment, low dosage of ATF inhibited tumour growth by blocking the proteolytic cascade initiated by uPA-uPAR interaction. Moreover, its antitumor effects could be further enhanced by TPL at a low dosage, suggesting a promising strategy to treat the devastating disease. During the development of colon cancer within nude mice, the tumour cells recruit murine endothelial cells to establish a network of new blood vessel. Human and mouse ATF are species specific. When entering into the circulation system, ATF was speculated to target only tumour cells instead of both the tumour and endothelial cells. Therefore, the anti-angiogenesis and antitumor activity may be partially compromised. We assumed that the antitumor function of ATF was achieved by its suppressive capacity against angiogenesis, which owes to its competitive interaction with uPAR towards uPA. In this case, TPL does not show species specific and can target both tumour and endothelial cells. Thus, when combined with TPL, ATF induced significantly increased antitumor and anti-angiogenesis efficiency. It is worth paying attention that only one colon cancer cell line was investigated in *in vivo* experiment in this study. More cancer cell lines are required to be studied *in vivo* to evaluate the therapeutic application of TPL and ATF combination on cancer in future.

## Conclusions

In summary, we provided evidence that TPL potently inhibited the growth of human solid tumour cell lines *in vitro*. We have also demonstrated that TPL, at a low concentration, synergistically induced cell apoptosis through multiple targets including caspases and NF-κB pathways in various tumour cell lines when combined with ATF (Figure 8). Furthermore, combined treatment with the two drugs effectively reduced growth of xenografted HCT116 cells grown in athymic mice without exhibiting any toxicity in the animals. Based on the synergistic antitumor activity profiles of combined TPL and ATF treatments *in vitro* and *in vivo* and the absence of cytotoxicity in normal tissues, we believe that TPL has strong therapeutic value for use in combination with ATF against colon cancer.

## Methods

### Cells, cell culture, and reagents

Human A549 lung adenocarcinoma cell line, human HCT116 colon cancer cell line, human breast cancer metastatic cell MDA-MB-231, human cervical carcinoma HeLa cell line, human embryonic renal HEK293 cells and human umbilical vein endothelial cells (HUVECs) were purchased from the American Type Culture Collection (ATCC, Philadelphia, PA, USA). MDA-MB-231, HeLa and HEK293 cells were grown in Dulbecco’s modified Eagle’s medium (DMEM) (HyClone, Logan, UT, USA) supplemented with 10% (v/v) fetal bovine serum (HyClone, Logan, UT, USA) and 1% penicillin-streptomycin (Invitrogen, Carlsbad, CA, USA). A549 and HCT116 cells were grown in RPMI 1640 (HyClone, Logan, UT, USA) supplemented with 10% (v/v) fetal bovine serum (HyClone, Logan, UT, USA) and 1% penicillin-streptomycin (Invitrogen, Carlsbad, CA, USA). HUVECs were grown in Medium 200 (Cascade Biologics, Portland, OR, USA) supplemented with Low Serum Growth Supplement (LSGS). All cells were cultured in a humidified CO_2_ incubator at 37°C. TPL (99% purity, Sigma-Aldrich, St. Louis, MO, USA) was solubilised in 0.01% dimethyl sulfoxide (DMSO) in phosphate buffered saline (PBS), filtered through a 0.2 μm Millipore filter and kept at −70°C. Annexin V and propidium iodide (PI) were purchased from Molecular Probes (Eugene, OR, USA).

### Expression and purification of ATF in *Pichia pastoris*

The plasmid pGAPZαA-*ATF* for the expression of ATF was constructed previously in our laboratory by Dr. Jianping Li. Plasmid DNA was then linearized at the *Bln* I site and electroporated into the yeast host strain X-33 (Invitrogen, Carlsbad, CA, USA). Recombinants were selected on YPDS plates (10 g/L yeast extract, 20 g/L peptone, 20 g/L dextrose, 20 g/L agar, and 1 M sorbitol) and characterized for expression of ATF. A single positive clone of Zeocin-resistant was selected with a view to produce the specific protein. A preculture growth step was performed for 24 h in a 250 mL Erlenmeyer flask containing 50 mL YPD medium. This cell culture was further used to inoculate larger yeast cell cultures at an optical density (OD_600 nm_) of 1, to start the cell growth directly in the exponential growth phase, as well as to establish reproducible cell culture conditions. The yeast was further grown at 30°C with orbital agitation at a rate of 250 rpm. The optimum YPD medium to flask volume ratio for ATF production was found to be 1/5 and the cultures were usually performed in a 1-L Erlenmeyer flask containing 200 mL YPD medium without any Zeocin. The cultures were stopped after 72 h and the cells were pelleted by centrifugation at 3000 × *g* for 20 min. Culture supernatants from shaker flasks were precipitated with ammonium sulphate (65% saturation). The precipitate was then dissolved in buffer A (20 mM sodium acetate, 1 mM EDTA, 0.5 mM PMSF, pH 4.8), and finally dialyzed against the same buffer at 4°C. Further purification was carried out by CM Sepharose Fast Flow column (GE Healthcare). After loading the sample, the column was washed with buffer B (20 mM sodium acetate, 1 mM EDTA, pH 4.8) and stepwise eluted by 0.1 M, 0.2 M, 0.5 M NaCl in buffer B. The eluted fractions were pooled and the concentration of ATF was determined by the Bio-Rad protein assay method (Bio-Rad, Hercules, CA). The purity was determined on a SDS-PAGE gel stained with Coomassie Blue. The identity of ATF was confirmed by Western blotting using polyclonal mouse anti-ATF antibody (Santa Cruz Biotechnology, Santa Cruz, CA).

### Cell proliferation assay

The effects of ATF, TPL or the combination on cell proliferation were assessed by the MTT assay. Cells in the exponential growth phase were seeded into a 96-well plate at a density of 5000 cells per well. After 24 h, ATF (10 nM), TPL (10 ng/mL) or the combination were added to the medium. The cells were incubated at 37°C for 24 h, then the cell viability was determined by the colorimetric MTT [3-(4, 5-dimethylthiazol-2-yl)-2, 5-diphenyl-2H-tetrazolium bromide] assay at wave length 570 nm by TECAN Safire Fluorescence Absorbance and Luminescence Reader (Vienna, VA, USA). The cell viability was calculated according to the formula: Cell viability (%) = average A_570__nm_ of treated group/average A_570 nm_ of control group × 100%. Each experiment was performed in quadruplicate and repeated at least three times. To determine whether TPL in combination with ATF worked synergistically, the combination index (CI) in MTT assay was calculated as follows: CI = AB / (A × B). According to cell viability of each treatment, AB is the ratio of the combination treatment to the control treatment; A or B is the ratio of the single agent treatment to the control treatment. Thus a CI value less than, equal to or greater than 1 indicates that the drugs are synergistic, additive or antagonistic, respectively. A CI less than 0.7 indicates that the drugs are significantly synergistic.

### Annexin V-fluorescein isothiocyanate/propidium iodide assay

To quantify the percentage of cells undergoing apoptosis, we used the Annexin V-FITC kit as described by the manufacturer. Briefly, HCT116 and A549 cells were incubated for 24 hours with TPL (10 ng/mL) and ATF (10 nM) alone or in combination. Next, the treated cells were collected and trypsinized for 3–5 min. The digested cells were washed twice with cold PBS and resuspended in binding buffer at a concentration of 1 × 10^6^ cells/mL. After incubation, 100 μL of the solution was transferred to a 5 mL culture tube, and 5 μL of Annexin V-FITC and 10 μL of PI were added. The tube was gently centrifuged and incubated for 15 min at room temperature in the dark. At the end of incubation, 400 μL of binding buffer was added, and the cells were analyzed immediately by flow cytometry (BD Biosciences, CA, USA). Flow cytometry analysis was performed with untreated HCT116 and A549 cells as control.

### Cell cycle analysis

HCT116 cells (2 × 10^6^) were treated with TPL (10 ng/mL) and ATF (10 nM) alone or in combination for 24 h. Cells were then harvested, washed in PBS, resuspended gently in 5 mL of 100% ethanol, and fixed at 25°C for 1 h. After washing with PBS, cells were incubated with DNase-free RNase A (200 μg/mL) at 37°C for 1 h and washed with PBS. PI (10 μg/mL) was added and the cells were incubated at 37°C for 5 min. The distribution of cells with differing DNA content was analyzed on a FACSCalibur flow cytometer with CellQuest software (BD Biosciences, CA, USA) at an excitation wavelength of 530 nm. Fluorescence emission was measured using a 620 nm band pass filter.

### Caspase activity assay

Caspase-3 and caspase-9 activities were measured using colorimetric activity assay kits (Chemicon International, CA, USA). The assay is based on the cleavage of the chromogenic substrates, DEVD-pNA and LEHD-pNA, by caspase-3 and caspase-9, respectively. Cells were lysed in chilled lysis buffer on ice for 10 min and centrifuged for 5 min at 10,000 × *g*. Caspase substrate solution containing the specific peptide substrate was then added to the supernatant and incubated for 2 h at 37°C before measurement by ELISA reader at 405 nm.

### RNA interference

The siRNA against NF-κB p65 (sc-29410) was purchased from Santa Cruz Biotechnology, Santa Cruz, CA. For transfection with siRNAs, logarithmically growing cells were transfected with siRNA as instructed by the manufacturer.

### Western blot analysis

HCT116 cells were incubated with TPL (10 ng/mL) and ATF (10 nM) alone or in combination for 24 h, then lysed with RIPA buffer (Beyotime, China) with protease inhibitor cocktail tablets (Complete Mini, EDTA free; Roche, Basel, Switzerland). Supernatants were collected and protein concentration was determined by the Bio-Rad protein assay method (Bio-Rad, Hercules, CA). Western blotting was performed according to standard protocols. Proteins were separated by SDS-PAGE and transferred onto nitrocellulose membranes that were blocked with 5% non-fat milk in TBS containing 0.1% Tween 20, and incubated with primary antibodies: p-FAK (Tyr 397), FAK, p-JNK, c-JUN, p-c-JUN, p-AKT (Ser 473), uPAR, cleaved caspase-3 (Cell Signalling Technology, Beverly, MA, USA), NF-κB p65 (Invitrogen, Carlsbad, CA), BAX, BAD, BAK, cIAP, poly (ADP-ribose) polymerase (PARP), α-tubulin, c-FLIP-L, GAPDH, Lamin B (Santa Cruz Biotechnology, Santa Cruz, CA). Secondary antibodies were coupled to horseradish peroxidase, and were goat anti-rabbit or goat anti-mouse. Bound antibodies were then visualized with ECL plus Western blotting detection reagents (GE Healthcare). Signal intensity was quantified by densitometry using the software Image J (NIH, Bethesda, MD). All experiments were done in triplicate and performed at least three times independently.

### RNA extraction and quantitative real-time PCR

Total RNA was extracted from treated cells using a TRIzol reagent (Invitrogen, Carlsbad, CA, USA) following the manufacturer’s instructions and was used to prepare cDNA by PrimeScript RT reagent Kit (Takara, Otsu, Shiga, Japan). Quantitative real-time PCR was performed with SsoFast EvaGreen Supermix on a CFX96 Real-Time System (Bio-Rad Laboratories, Hercules, CA, USA). The sequences of PCR primers used in our study were synthesized commercially, and are shown as follows: MMP-2 upstream: 5′ -CCGTCGCCCATCATCAA- 3′; MMP-2 downstream: 5′ GGTATTGCACTGCCAACTCTTTG- 3′; MMP- upstream: 5′ GGACGATGCCTGCAAGT- 3′; MMP-9 downstream: 5′ -ACAAATACAGCTGGTTCCCAATC- 3′. The glyceraldehyde 3-phosphatase dehydrogenase (GAPDH) gene was used as the reference gene. All data were means of fold change of triplicate analysis and normalized with those of GAPDH.

### Cell migration assay

The effects of ATF, TPL or the combination on endothelial cell and tumor cell migration were assessed by the transwell assay. The cell migration assay was performed using transwell inserts (8.0 mm pore size, Millipore, Billerica, MA, USA) as described previously [71]. Before the experiment, HUVECs and HCT116 cells had been cultured in serum-free medium with ATF (10 nM), TPL (10 ng/mL) or the combination (PBS used as buffer control) for 16 h. Then the cells were harvested and resuspended in the same medium. 1 × 10^5^ cells in a volume of 0.1 mL were added to the upper chamber, and the lower chamber was filled with 0.6 mL of 20% FBS supplemented medium. After incubation at 37°C for 9 h, cells on the upper surface of the membrane were removed. The migrant cells attached to the lower surface were fixed in 10% formalin at room temperature for 30 min, and stained for 20 min with a solution containing 1% crystal violet and 2% ethanol in 100 mM borate buffer (pH 9.0). The number of cells migrating to the lower surface of the membrane was counted in five fields under a microscope with a magnification of × 100. All groups of experiments were conducted in triplicate, and the cell number was counted by Image-Pro Plus 6.0 software.

### *In vivo* animal tumour model experiment

Athymic nude mice (6–8 weeks of age) were obtained from Shanghai Laboratory Animal Centre (Shanghai, China) and housed under germfree conditions. Animal care and use were performed strictly in accordance with the ethical guidelines by Nanjing University Animal Care and Use Committee and the study protocol was approved by the local institution review board. HCT116 cells (2 × 10^6^ cells in 50 μL) were injected subcutaneously into the dorsal flanks of mice. Tumour volume was monitored by measuring the two maximum perpendicular tumour diameters with callipers every alternate day. All tumour-bearing mice were divided randomly into 4 groups, and treatment was initiated on the 7th day when the volume of tumour reached a size of approximately 50 mm^3^. The mice were injected intraperitoneally (i.p.) with ATF (100 nmol/kg), TPL (100 μg/kg) or the combination every two day for a total of 21 day. Control mice received i.p. injection of PBS. Antitumor activity of treatments was evaluated by tumour growth inhibition. Tumours were measured individually with a calliper every other day, and the formula, tumour volume = length × width^2^ × 0.52 was used to mimic the tumour volume. At the end of study, the tumours were collected and weighed.

In a parallel animal assay (totally 4 groups, and 3 mice per group), the tumour establishment and drug treatment are the same as described earlier. On the 21th day, mice were euthanized. Tumours were collected, fixed with 4% formaldehyde, embedded in paraffin and sectioned for haematoxylin and eosin (H&E) staining or immunostaining according to standard histological procedures. Blood vessel within tumours was immunostained with anti-mouse CD31 monoclonal antibody (BD Pharmingen, Franklin Lakes, NJ, USA) and determined by the average number of vessels in 3 regions of highest density at 200 × magnifications in each section.

### Calculation of tumour doubling time and combination index

The tumour doubling time (TDT) and combination index (CI) were calculated using GraphPad Prism v 5.0. TDT values were generated from exponential growth curves, which had been fitted to % change in tumour volume data (*r*^2^ > 0.70). Our CI calculations were adapted [72] to apply to TDT values. First, the TDT value for untreated mice was subtracted from the TDT value for each treatment group to obtain ‘blanked’ TDT values (TDT_B_). Then, the CI was calculated as the ratio of TDT_B_ values of combination treatment to individual treatments: CI = (TDT_B_ combination of TPL and ATF)/(TDT_B_ TPL alone + TDT_B_ ATF alone).

### Statistical analysis

Statistical analysis was carried out using the SPSS software (version 11.0; SPSS, Chicago, IL). Data were expressed as the mean ± standard deviations (SD) and analyzed by one-way ANOVA and the least significant difference tests. *P* < 0.05 was considered statistically significant.

## Competing interests

The authors declare that they have no competing interests.

## Authors’ contributions

ZCH and HZ designed the study; YL, NP and JL performed experiments; YL and HZ analyzed data; HZ and ZCH wrote the paper. All authors read and approved the final manuscript.
